# Educators’ Beliefs and Motivational Orientations as Predictors of ICT Use in Early Childhood Education

**DOI:** 10.3390/bs16071219

**Published:** 2026-07-18

**Authors:** Franziska Cohen, Theresia G. Hummel, Yvonne Anders

**Affiliations:** 1 Department of Early Childhood Education, University of Education Freiburg, Kunzenweg 21, 79116 Freiburg, Germany; 2 Department of Early Childhood Education, University of Graz, 8010 Graz, Austria; 3 Department of Child Development in Educational Contexts, DIPF—Leibniz Institute for Research and Information in Education, 60323 Frankfurt am Main, Germany

**Keywords:** early childhood education, digital technologies, ICT integration, educators’ beliefs, self-efficacy

## Abstract

Digital technologies are an integral part of young children’s everyday lives, yet their pedagogical integration in early childhood education and care (ECEC) remains limited and highly variable across ECEC centers. Drawing on the extended Structure–Process (SP-E) framework, this study examines how structural characteristics of ECEC centers, particularly the range of available ICT equipment, and educators’ ICT-related professional competencies, especially beliefs and motivational orientations, predict ICT implementation in pedagogical practice. Data from the DIGIPaed project comprised 266 educators across 97 German ECEC centers. Hierarchical regression models with cluster-adjusted standard errors were estimated for two outcomes: ICT use with children and pedagogical ICT activities. Results revealed distinct predictor patterns for the two outcomes. The range of available ICT equipment was positively associated with pedagogical ICT activities but not with ICT use with children. ICT-related self-efficacy was the only professional competence variable to predict both outcomes, explaining additional variance beyond structural conditions and beliefs. Centers with higher proportions of children with a migration background showed lower levels of ICT use with children. These findings suggest that ICT implementation in ECEC is not a unitary construct and that different dimensions of implementation may depend on different conditions. Self-efficacy emerged as a particularly promising target for professional development aimed at supporting meaningful ICT integration in ECEC centers.

## 1. Introduction

### 1.1. ICT in Early Childhood Education

Digital technologies are increasingly present in young children’s everyday environments. Institutions of early childhood education and care (ECEC centers) are expected to pedagogically engage with children’s living realities and therefore with their existing digital experiences (Lähdesmäki et al., 2023; Undheim, 2021). Evidence suggests that ICT^1^ can support early learning, particularly in the domains of language development and early literacy (Egert et al., 2022; S. Liu et al., 2024; Nwachukwu et al., 2025). As in other domains of early childhood education (Anders, 2013; Burger, 2010), the quality of pedagogical practice appears to be the key factor in determining whether ICT contributes meaningfully to children’s learning opportunities. Studies show that benefits are realized when implementation demonstrates high quality. In this context, the active roles of educators and children, as well as opportunities for interaction, are crucial (Cordes et al., 2023; S. Liu et al., 2024).

Despite this potential, pedagogical ICT integration in ECEC varies considerably across ECEC centers. In Germany, centers (i.e., publicly funded day care centers for children aged zero to six) range from deliberately analog to dedicated media-focused institutions (Brüggemann, 2018), and empirical studies document broad heterogeneity in ICT use across centers (Cohen et al., 2022; Knauf, 2019). Where ICT is used, it predominantly serves documentation, administration, and parent communication rather than children’s learning (Hummel et al., 2025; Knauf, 2020; Pfützner & Knauf, 2023; Restiglian et al., 2023). This discrepancy between the documented potential of ICT and its limited pedagogical use points to persistent challenges in implementation.

Research on technology integration distinguishes first-order barriers, such as lack of infrastructure, time constraints, and limited institutional support, from second-order barriers rooted in educators’ professional competencies (Ertmer, 1999). In Germany, surveys indicate that while most centers have internet access, only around two-thirds have WLAN used for pedagogical activities, tablets are present in fewer than half of centers, and between a third and half of educators rate their ICT equipment as insufficient (Stiftung Haus der kleinen Forscher, 2017; Anders et al., 2022; Cohen et al., 2022). Lack of infrastructure is among the most consistently reported barriers to broader ICT integration in ECEC (Akram et al., 2022; Kabadayi et al., 2022; X. Liu & Pange, 2015; Magen-Nagar & Firstater, 2019). Even though structural conditions may limit practical applications, they do not entirely determine implementation. Whether available technologies are used for educational purposes depends largely on individual-level factors (Mumtaz, 2000). Such second-order barriers include educators’ beliefs about the value of technology, their confidence in using it, and their affective dispositions toward ICT (Ertmer, 1999; Ertmer & Ottenbreit-Leftwich, 2010). Although a growing body of research has examined how these individual factors relate to ICT use in educational settings, much of this work has focused on technology-related knowledge (e.g., Blackwell et al., 2016) or general attitudes (e.g., Konca et al., 2016; Sivropoulou et al., 2009). Less attention has been paid to the specific role of motivational orientations such as self-efficacy, enthusiasm, and anxiety as predictors of ICT implementation in ECEC. Yet these constructs are particularly relevant because they are more proximal to professional behavior than general attitudes and, unlike formal qualifications, are potentially modifiable through professional development (Bandura, 1997; von Suchodoletz et al., 2018). The present study addresses this gap by examining both structural conditions and a differentiated set of professional competencies within a single analytical framework.

### 1.2. The Extended Structure–Process (SP-E) Framework

Examining both structural and individual-level characteristics within a single model requires a theoretical framework that integrates these two levels. Building on prior research on first- and second-order barriers, this study adopts the extended Structure–Process (SP-E) framework (Anders & Oppermann, 2024), which was developed for the ECEC context and conceptualizes how institutional conditions and educators’ competencies jointly shape pedagogical ICT use. The SP-E framework builds on the established structure–process model of ECEC quality (Kluczniok & Roßbach, 2014), which distinguishes structural quality from orientation quality and process quality and links all three quality dimensions to children’s developmental outcomes. In the extended SP-E framework, the latter dimension is conceptualized more specifically as interaction quality, emphasizing the quality of pedagogical interactions between educators and children (Anders & Oppermann, 2024). Further, the extended model integrates additional theoretical strands and distinguishes dimensions of ECEC quality that existing models had addressed separately. Structural quality refers to the organizational and material conditions of ECEC centers, including staff qualifications, group size, staff–child ratios, material resources, and group composition. Interaction quality refers to the quality of educational interactions and learning activities that children directly experience. It represents the proximal dimension, which is most strongly linked to developmental outcomes. Interaction quality can be further differentiated along two dimensions. First, a distinction exists between the quantity or frequency of educational activities and their qualitative characteristics, such as intentionality and developmental appropriateness. Second, interaction quality can be conceptualized at a global level, capturing the overall quality of educator–child interactions, or at a domain-specific level, reflecting practices within particular content areas such as language, mathematics, or digital media (Anders, 2013; Kluczniok & Roßbach, 2014). The present study draws on this differentiation by examining two ICT-related outcomes that reflect different aspects of implementation: the frequency of device use with children (a quantitative indicator) and the extent to which educators engage children in intentional, pedagogically focused digital media activities (a more qualitative, domain-specific indicator).

The extended model further incorporates educators’ professional competencies as an explicit dimension, conceptualized as the mechanism through which structural characteristics are interpreted and translated into pedagogical processes. Therefore, structural characteristics function as enabling or constraining conditions for pedagogical practice. While they do not shape children’s experiences directly, they determine the opportunities and limitations within which interaction quality unfolds. The model of professional competence underlying the SP-E framework is based on Baumert and Kunter’s (2011) conceptualization of teacher professionalism as a multidimensional construct comprising professional knowledge, beliefs, motivational orientations, and self-regulatory skills. This model was originally developed in the context of school education and later adapted to the ECEC context (Anders, 2012). In the present study, motivational orientations are conceptualized broadly to include not only intrinsic motivation (captured here as ICT enthusiasm) and self-efficacy, both of which are explicitly represented in the Baumert and Kunter model, but also ICT anxiety as an affective–motivational cost component (Wigfield & Eccles, 2000). While anxiety is not part of the original professional competence model, its inclusion is justified by theoretical and empirical links to self-efficacy and technology adoption behavior (Beckers & Schmidt, 2001).

The professional competencies of early childhood educators, including their beliefs, self-efficacy, and affective dispositions, are thus key determinants of interaction quality. Applied to ICT implementation, the SP-E framework implies that the pedagogical use of digital technologies cannot be reduced to the availability of devices. Rather, it is shaped by the interplay of ICT-related structural conditions and educators’ professional competencies, which determine whether and how those resources are used in pedagogical practice.

In this study, structural characteristics include the range of available ICT equipment and WLAN access as material resources. Group composition variables, specifically staff–child ratio, proportion of children under three, and proportion of children with a migration background, are included as structural controls. These variables are relevant because they constrain pedagogical capacity and priorities. The staff–child ratio limits the time for planned activities; groups with high proportions of children under three may prioritize care over ICT use (Rettenbacher et al., 2022); and centers with higher proportions of children with a migration background tend to show lower structural and process quality (Lehrl et al., 2014; Stahl, 2015), which may affect ICT-related resources and pedagogical priorities.

### 1.3. Educators’ Professional Competencies and ICT Implementation

While frameworks such as DigCompEdu (Redecker, 2017) characterize the competence dimensions required for educators’ professional technology use, the present study complements this perspective by focusing on how ICT-related beliefs, self-efficacy, and motivational orientations shape pedagogical practice.

Whether educators use available ICT for educational purposes depends in part on their personal beliefs regarding the appropriateness and usefulness of digital technology in ECEC. Such ICT-related beliefs, understood as experience-based orientations that shape how individuals perceive and respond to professional situations (Fives & Buehl, 2012; Ertmer, 2005), capture educators’ views on whether digital media are developmentally suitable and beneficial for young children. These beliefs encompass views on the pedagogical value of ICT across developmental domains and guide as implicit filters pedagogical decision making (Perren et al., 2017). Positive ICT beliefs are associated with more frequent integration of ICT in daily pedagogical practice (Blackwell et al., 2014; Kerckaert et al., 2015; Nikolopoulou & Gialamas, 2015). Where ICT is viewed as a developmental risk, a replacement for play, or as developmentally inappropriate, pedagogical use tends to be restricted or avoided (Mertala, 2017). However, the relationship between beliefs and pedagogical practice is not always consistent. Dong (2018), for example, found that preschool teachers held highly positive perceptions of ICT benefits and still rarely employed pedagogical strategies to support children’s ICT use, which means that positive beliefs are not necessarily transferred to pedagogical practice. In the ECEC context, this tension is particularly pronounced. Skepticism toward the use of digital media in early childhood is not only a barrier to implementation but also reflects legitimate pedagogical concerns regarding the developmental appropriateness of ICT use (Palaiologou, 2016). Whether a positive attitude toward ICT should be understood as a prerequisite for high-quality implementation, or whether a critical perspective can also serve a protective function, remains unclear.

ICT-related self-efficacy beliefs refer to educators’ perceived competence to purposefully integrate digital technologies into pedagogical practice with young children. Following Bandura (1997), self-efficacy is domain-specific. It concerns what one believes one can do in a particular situation, not generalized confidence. Further, self-efficacy beliefs are understood as the perceived ability to successfully manage challenging or novel tasks in a specific domain. In the ECEC context, this includes confidence in planning, implementing, and reflecting on ICT-based learning activities (Blackwell et al., 2014; Nikolopoulou & Gialamas, 2015; Valdez et al., 2025).

Self-efficacy is conceptually distinct from ICT-related beliefs. Beliefs concern the perceived value of ICT as a pedagogical tool. Self-efficacy concerns perceived competence to use it. An educator may view digital media as beneficial for children’s learning yet lack the confidence to plan and implement such activities. Both constructs independently predict ICT implementation in educational settings (Blackwell et al., 2014; Ertmer & Ottenbreit-Leftwich, 2010; Mlambo et al., 2020). According to Bandura (1997), self-efficacy is shaped by prior experience and social learning, making it potentially responsive to professional development (von Suchodoletz et al., 2018). It should be noted that self-efficacy is sometimes classified as a belief construct (Bandura, 1997, uses the term “self-efficacy beliefs”), while in the theoretical tradition of professional competencies, it is treated as a component of motivational orientations (Baumert & Kunter, 2011). The present study follows the latter tradition and subsumes self-efficacy under the broader category of motivational orientations, alongside enthusiasm and anxiety.

Alongside beliefs and self-efficacy, this study examines ICT enthusiasm and ICT anxiety as affective–motivational orientations. Using Eccles’ expectancy-value model (Wigfield & Eccles, 2000), motivational orientations toward a given task can be understood as comprising an expectancy component (beliefs about one’s likelihood of success) and a value component, which includes intrinsic interest and perceived cost. Applied to ICT implementation, self-efficacy captures the expectancy dimension, while enthusiasm reflects intrinsic value and positive affect associated with digital technology use in professional contexts (Davis, 1989). Additionally, anxiety reflects the emotional cost associated with technology use (Beckers & Schmidt, 2001). Whether enthusiasm and anxiety predict ICT implementation independently of beliefs and self-efficacy remains an open empirical question in the ECEC context (Dong & Mertala, 2021; Murcia et al., 2018).

## 2. Research Questions and Hypotheses

Against the background of the SP-E framework outlined above, several gaps in the current literature on ICT implementation in ECEC were identified. Structural conditions and individual-level factors have rarely been examined within the same analytical model. Motivational constructs such as self-efficacy, enthusiasm, and anxiety have received limited attention as distinct predictors beyond general beliefs. And few studies have differentiated between aspects of ICT implementation that may be predicted by different factors. The present study addresses these gaps. Two conceptually distinct outcomes are examined: (1) ICT use with children and (2) pedagogical ICT activities. Both are expected to be predicted by the same set of structural and competence-related factors. However, because the two outcomes capture qualitatively different aspects of ICT implementation, one reflecting the frequency of device use and the other the intentionality of pedagogical practice, differential predictive patterns are possible.

RQ1: To what extent are structural characteristics of ECEC centers associated with ICT implementation? H1: The range of available ICT equipment is positively associated with ICT implementation.

RQ2: How are educators’ ICT-related beliefs associated with ICT implementation? H2: Positive ICT-related beliefs are positively associated with ICT implementation.

RQ3: How far do motivational orientations, specifically self-efficacy, enthusiasm, and anxiety, contribute additional effects beyond structural conditions and beliefs? H3: ICT-related self-efficacy explains additional variance in ICT implementation beyond structural conditions and beliefs. No directional hypotheses are specified for enthusiasm and anxiety, as prior evidence on their independent contributions in the ECEC context is insufficient to formulate differentiated predictions.

## 3. Analytical Framework

**Figure 1:**
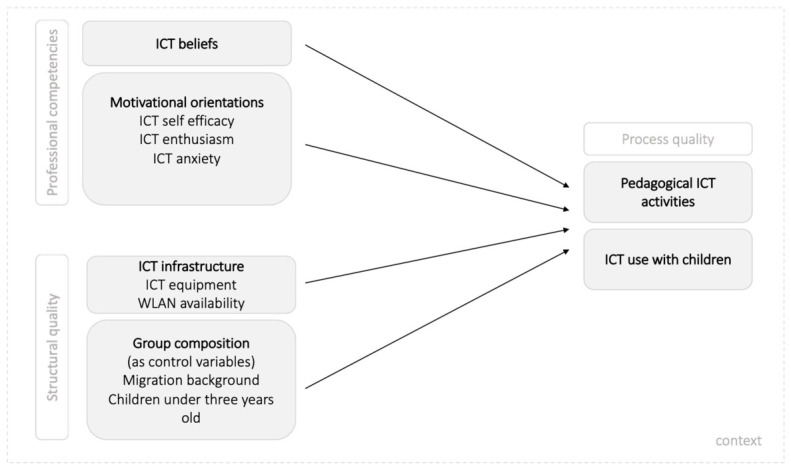
Analytical Framework of Structural and Individual Predictors of ICT Implementation in Early Childhood Education. Note. The model draws on the extended Structure–Process framework (Anders & Oppermann, 2024). Structural characteristics and professional orientations are hypothesized to jointly predict two dimensions of ICT implementation in early childhood education.

To visualize how the theoretical constructs outlined above are operationalized in the present study, Figure 1 shows the analytical framework. Based on the SP-E model (Anders & Oppermann, 2024), structural characteristics and educators’ professional competencies (ICT beliefs, self-efficacy, enthusiasm, anxiety) are examined as predictors of ICT implementation. In this study, ICT implementation encompasses both the frequency with which educators use digital devices together with children and the extent to which they engage children in intentional, pedagogically focused digital media activities. Structural conditions are conceptualized as enabling factors that set the material prerequisites for ICT use, while professional competencies represent the individual-level orientations that shape whether and how those conditions are drawn upon in pedagogical practice with children. Structural characteristics include range of available ICT equipment and WLAN access as the primary predictors of interest, alongside group composition variables included as structural controls: staff–child ratio, proportion of children with migration background, and percentage of children under three years of age.

## 4. Methods

### 4.1. Study Design and Sample

The present study analyzed data from the DIGIPaed project, which investigated digital media use in ECEC centers in Germany (Cohen & Anders, 2023; Cohen et al., 2022). The study is based on a cross-sectional design. To ensure a balanced sample of urban and rural areas, as well as centers in Western and Eastern Germany, data were collected randomly across four German states (Bavaria, Bremen, North Rhine-Westphalia, and Saxony-Anhalt). Data collection took place between September 2020 and August 2021. A total of 266 educators from 97 centers (on average 2.8 educators per center) completed a standardized questionnaire covering ICT availability, pedagogical uses of digital media, and professional orientations toward technology. Participating ECEC centers serve children from birth to school entry (age 0–6), and the mean center size was 76 children (SD = 34, range 15–198). All three forms, age-heterogeneous groups (children aged 0–6), groups with only preschool-age children (3–6), and groups with only children under 3 years of age, were represented in the sample, reflected by the inclusion of the proportion of children under three years of age as a structural control variable. Participation was voluntary. All participants provided informed consent; no personally identifiable information was collected, and all data were analyzed in anonymized form.

### 4.2. Measures

#### 4.2.1. ICT Implementation

Two dependent variables were constructed. *ICT use with children* was operationalized as a summative formative index, adding educators’ self-reported frequency of ICT use across six types of devices (digital cameras, tablets, computers/laptops, smartphones, projectors, and digital toys) on a six-point scale (1 = never, 6 = daily; theoretical maximum: 36) (Then et al., submitted). A six-point format was used to differentiate between usage frequencies ranging from never to daily without offering a neutral midpoint. The index captures a cumulative device engagement rather than a single underlying latent construct. Internal consistency coefficients are therefore not applicable and are not reported.

*Pedagogical ICT activities* were assessed as a summative index based on two items on a six-point scale (1 = never, 6 = daily; theoretical maximum: 12) (Six & Gimmler, 2007, Then et al., submitted): “the use of media as a tool for creative expression and production”, and “the use of ICT as a tool in educational activities, but the medium itself is not the primary focus”. These two items were selected because they capture active, intentional ICT engagement in which digital tools are directly employed in educational interactions with children. The remaining items from the original instrument (e.g., media use for relaxation, discussing media experiences) reflect broader media education practices that do not necessarily involve active ICT use and were therefore not included. The construct was treated as a formative index, as well.

Together, the two outcome variables capture complementary aspects of ICT implementation: ICT use with children reflects how frequently different device types are used in everyday practice, while pedagogical ICT activities capture the extent to which educators engage children in intentional, educationally focused digital media activities. The former is a quantitative indicator of device engagement, while the latter is a more qualitative indicator of pedagogical intentionality.

#### 4.2.2. Professional Competencies

*ICT beliefs* were assessed using a 21-item mean scale covering educators’ reported attitudes toward the educational value of digital technologies across developmental domains (cognitive, creative, social-emotional, motor, intercultural) on a four-point scale (1 = I don’t agree, 4 = I agree; α = .91; example item: “Digital media helps children develop their problem-solving skills”). Higher scores indicate more positive beliefs. Items were drawn from existing validated scales (Gialamas & Nikolopoulou, 2010; Nikolopoulou & Gialamas, 2015; Konca et al., 2016; Sivropoulou et al., 2009; Preradóvić et al., 2017; Pfost et al., 2018) and supplemented by newly developed items. *ICT self-efficacy* was measured with a mean scale of three items assessing educators’ perceived competence to plan, implement, and reflect on digital learning activities with children (1 = I don’t agree to 4 = I agree, α = .81; example item: “I’m confident that I can integrate digital media into educational activities in a way that helps children promote their learning”). Items were adapted from Nikolopoulou and Gialamas (2015); additional items were developed for the present study. *ICT enthusiasm* captured positive affect and intrinsic motivation of the educator when engaging with digital technologies in educational contexts on a four-point scale with three items (1 = I don’t agree to 4 = I agree, α = .87; example item: “Teaching children how to use digital media is something I’m passionate about”). *ICT anxiety* captured fear and discomfort of the educator associated with the use of digital tools in educational situations with children. The four-point scale (1 = I don’t agree to 4 = I agree; example item: “Helping children use digital media in their daily lives can be stressful for me”) comprised four items, with higher scores indicating greater anxiety (α = .77). Anxiety items were developed for the present project, adapted in structure from the Mathematics Anxiety Scale-Revised (Bai et al., 2009) and adjusted to the ICT context.

#### 4.2.3. Structural Characteristics

The *ICT equipment* in the center was operationalized as a count-based index capturing the range of digital device types available for use with children. *WLAN availability* was operationalized as a dichotomous indicator of wireless internet access in the classroom. The *staff–child ratio,* the *proportion of children with a migration background (%)*, and the *percentage of children under three years of age* reported by the teacher were included as control variables.

### 4.3. Analytical Strategy

Three hierarchical regression models were estimated. Model 1 included structural characteristics, model 2 added ICT-related beliefs, and model 3 further included motivational orientations (self-efficacy, enthusiasm, and anxiety). Because educators were nested within centers, cluster-adjusted standard errors were applied across all models to account for the non-independence of observations using TYPE = COMPLEX in Mplus 8 (Muthén & Muthén, 2023). The intraclass correlations indicated moderate clustering in the use of ICT when working with children (ICC = .149) and very low clustering in pedagogical ICT activities (ICC = .039).

Missing data ranged from 6% to 18% across study variables. Preliminary analyses indicated that data were not missing completely at random (MCAR) and were therefore handled using Full Information Maximum Likelihood (FIML) estimation, which provides unbiased parameter estimates under the missing at random (MAR) assumption (Enders & Bandalos, 2001; Lüdtke et al., 2007).

Sensitivity power analysis (G*Power 3.1, Faul et al., 2009) indicated adequate power to detect small-to-medium effects (f^2^ = 0.10; N ≈ 209 adjusted for clustering; k = 9; α = .05; power = .90). Variance inflation factors (VIFs) computed in supplementary OLS regressions in Stata SE 17 ranged from 1.03 to 2.27 (M = 1.39), indicating no serious multicollinearity concerns among predictors.

## 5. Results

Descriptive statistics, reliability coefficients, and intercorrelations for all study variables are presented in Table 1 and Table 2. Both outcome variables were situated in the lower range of their respective scales: mean ICT use with children was 3.89 (SD = 1.14) against a theoretical maximum of 36, and mean pedagogical ICT activities was 4.30 (SD = 2.28) against a theoretical maximum of 12. Regarding the predictor variables, the mean ICT equipment score of 2.68 (SD = 1.46) indicates that centers had, on average, between two and three types of devices available for educational work, suggesting limited equipment breadth in many centers. This low baseline may contribute to the overall low levels of ICT use observed. ICT beliefs (M = 2.78, SD = 0.47) and ICT self-efficacy (M = 2.84, SD = 0.70) were moderately positive, while ICT anxiety was low (M = 1.47, SD = 0.57). Notably, the standard deviation of pedagogical ICT activities (SD = 2.28) was proportionally larger than that of ICT use with children (SD = 1.14), indicating greater variability in intentional pedagogical ICT practices across educators despite the narrower theoretical range.

### 5.1. ICT Use with Children

The first research question addressed whether structural characteristics and professional competencies predict ICT use with children. Results are presented in Table 3. Among the structural controls, the proportion of children with a migration background showed a consistent negative association across all three models (β = −.18 to −.20, *p* < .05). No other structural predictor reached significance. Structural characteristics explained 5.0% of the variance. ICT beliefs did not significantly predict ICT use with children in Model 2 (β = −.07, *p* > .10; R^2^ = .055). In Model 3, ICT self-efficacy was positively associated with ICT use with children (β = .19, *p* = .025), while ICT beliefs showed a marginally significant negative association (β = −.17, *p* = .052). ICT enthusiasm (β = .08, *p* = .467) and ICT anxiety (β = −.06, *p* = .516) were non-significant. Model 3 explained 11.6% of the variance.

### 5.2. Pedagogical ICT Activities

The second research question examined the same predictors in relation to pedagogical ICT activities. Results are presented in Table 4 and show a different pattern compared to ICT use with children. In Model 1, ICT equipment showed a positive association with pedagogical ICT activities (β = .17, *p* = .034); the proportion of children with a migration background showed a marginal negative association (β = −.12, *p* = .058). WLAN availability, staff–child ratio, and percentage of children under three were non-significant. Structural characteristics explained 4.7% of the variance. Adding ICT beliefs in Model 2 increased the explained variance to R^2^ = .087; positive associations emerged between beliefs and the pedagogical activities (β = .20, *p* = .002), while ICT equipment (β = .16, *p* = .044) and migration background (β = −.13, *p* = .036) remained significant. In Model 3, ICT self-efficacy showed a significant positive association with pedagogical ICT activities (β = .20, *p* = .015), while the association between beliefs and the outcome was reduced and no longer significant (β = .10, *p* = .164). Neither ICT enthusiasm (β = .12, *p* = .251) nor ICT anxiety (β = .13, *p* = .153) reached significance. ICT equipment remained significant (β = .16, *p* = .032). Model 3 explained 14.4% of the variance.

## 6. Discussion

Digital technologies are increasingly present in young children’s everyday lives, yet their educational integration in ECEC centers remains limited. The present study examined structural characteristics of ECEC centers and educators’ ICT-related professional competencies as predictors of two dimensions of ICT implementation. Against the theoretical background of the extended structure-process model (Anders & Oppermann, 2024), three research questions guided the analysis, addressing the predictive role of structural conditions, ICT-related beliefs, and motivational orientations such as self-efficacy, enthusiasm, and anxiety. Results indicated that the range of available ICT equipment showed consistent positive associations with pedagogical ICT activities but no significant association with ICT use with children. H1 is therefore supported only partly. Self-efficacy emerged as the most consistent predictor of both outcomes, while beliefs, enthusiasm, and anxiety did not independently contribute once self-efficacy was controlled for. H2 received partial support, because ICT beliefs were positively associated with pedagogical ICT activities when entered alone in Model 2, but this association was no longer significant once self-efficacy was included in Model 3. No significant association emerged for ICT use with children. H3 was supported across both outcomes, with self-efficacy explaining additional variance beyond structural conditions and beliefs. Beyond the stated hypotheses, the proportion of children with migration background emerged as a consistent negative predictor of ICT use with children, a pattern discussed below.

### 6.1. Structural Conditions

The range of available ICT equipment showed consistent positive associations with pedagogical ICT activities but was unrelated and directionally negative for ICT use with children. Thus, H1 was partially confirmed, consistent with the interpretation that the two outcomes reflect qualitatively different dimensions of ICT implementation. A greater range of available device types may open up more diverse pedagogical possibilities, enabling educators to plan activities that draw on different technologies for different purposes. In this interpretation, equipment breadth functions as an enabler of pedagogical complexity. Implementing a planned digital storytelling session with a group of children, for example, requires a tablet or interactive display that is readily accessible during activities. ICT use with children, by contrast, reflects a broader range of device-specific practices that may be less contingent on infrastructure availability. Briefly using a smartphone to photograph a child’s construction or playing a short video clip requires no particular equipment variety and may occur even in centers with limited digital resources. The frequency of using individual devices may thus be driven more by personal routines and perceived competence than by the variety of available resources, which would explain why self-efficacy but not equipment predicted ICT use with children.

The fact that self-efficacy, but not equipment, showed associations with both outcomes further supports this interpretation. Perceived competence was associated with ICT engagement across both forms of implementation, whereas material resources were primarily associated with intentional, educationally oriented activities. This is consistent with Kerckaert et al. (2015), who found that ICT infrastructure predicted curriculum-oriented technology use in primary education. The present study extends this finding by showing that the association holds for intentional pedagogical activities but not for general device use. It should also be noted that the availability of equipment does not necessarily imply that educators receive the technical or pedagogical support needed to use it effectively. Access to functioning devices and the availability of ongoing support for their integration into practice are distinct conditions that the present study could not differentiate.

WLAN availability was unrelated to either outcome. This may reflect that WLAN primarily serves as a basic prerequisite for internet-dependent applications rather than as a differentiating factor for pedagogical ICT use. Moreover, several common devices used in ECEC centers, such as digital cameras, projectors, or preloaded tablets, can be used offline and do not require wireless connectivity.

The proportion of children with a migration background was the only structural predictor to remain significant after professional competence variables were included in the models, and only for ICT use with children. A marginal negative association with pedagogical ICT activities points in the same direction, though it did not reach conventional significance. This pattern may reflect two distinct pathways. Centers with higher proportions of children with migration background tend to show lower structural and process quality more broadly (Lehrl et al., 2014; Stahl, 2015), a pattern that has also been observed specifically for ICT-related process quality (Schroeter et al., submitted), which may limit the material resources available for ICT use with children. Beyond resource constraints, such centers may also face higher pedagogical demands around language support and social integration that compete with ICT integration for time and attention, reducing the priority of digital activities in everyday practice. The cross-sectional design does not allow for causal conclusions at this point. However, this finding has implications that extend beyond the implementation of ICT in the ECEC context. If children in centers with a higher proportion of children with a migration background are systematically offered fewer ICT-supported learning opportunities, this can be an additional dimension of educational disadvantage that further increases existing inequalities in access to high-quality early childhood education. Early childhood education has been identified with the potential to bridge digital inequalities between sociodemographic groups (OECD, 2023). However, the present findings suggest that this potential is not yet being fully realized and that the digital divide in ECEC could be reproduced not only by unequal infrastructure but also by the unequal distribution of pedagogically meaningful ICT use across different groups of children.

### 6.2. Beliefs and Self-Efficacy

ICT beliefs showed significant positive associations with pedagogical ICT activities in Model 2. However, this association was reduced and became no longer significant once self-efficacy was included in Model 3. Thus, H2 received only partial support, while H3 was supported regarding both outcomes, as self-efficacy explained additional variance beyond structural conditions and beliefs. This pattern is consistent with established models of teacher cognition, which conceptualize beliefs as distal orientations that are less directly linked to pedagogical behavior than more proximal constructs such as self-efficacy (Muijs & Reynolds, 2002; Fives & Buehl, 2012). From this perspective, beliefs are assumed to shape behavior indirectly, whereas self-efficacy exerts a more immediate influence on instructional decision-making. The present findings are therefore theoretically plausible. Earlier studies in ECEC have identified self-efficacy as one of the strongest predictors of ICT adoption (Blackwell et al., 2014; Nikolopoulou & Gialamas, 2015). The present findings extend this work by showing that self-efficacy predicts not only general device use but also intentional pedagogical activities. Whether this pattern applies to ICT implementation behavior in ECEC remains an open question that requires longitudinal data to address.

An additional finding deserves methodological consideration. ICT beliefs showed a marginally significant negative association with ICT use with children in Model 3 (β = −.17, *p* < .10), despite a positive bivariate correlation (r = .28, *p* < .01; Table 2). This is an indicator of a statistical suppression effect, in which the inclusion of correlated predictors, particularly self-efficacy, changes the direction of an individual coefficient. Given the shared variance between beliefs and self-efficacy (r = .23) and between beliefs and enthusiasm (r = .60), the negative coefficient is more likely a statistical artifact of shared variance among predictors than a substantive effect of beliefs on ICT use. It is also conceivable that educators with both strong beliefs and high self-efficacy concentrate their ICT use on intentional, pedagogically oriented activities rather than on frequent general device use, but this interpretation remains speculative and cannot be tested with the present data.

### 6.3. Enthusiasm and Anxiety

ICT enthusiasm and ICT anxiety were not significant predictors of either outcome in the final models, although correlations were present (Table 2). Although variance inflation factors indicated no serious multicollinearity (VIF ≤ 2.27), ICT enthusiasm shares substantial variance with both beliefs (r = .60) and self-efficacy (r = .53). Its non-significance in the final model may therefore reflect conceptual overlap rather than irrelevance as a predictor of ICT implementation. This pattern is consistent with Bandura’s (1997) concept of the development of self-efficacy. According to this, affective and emotional states are sources of self-efficacy. From this perspective, enthusiasm and anxiety may indirectly shape ICT-related practice through their influence on perceived competence. Longitudinal studies are needed to investigate whether enthusiasm and anxiety predict the development of self-efficacy among professionals over time and whether their relationships with practice are mediated by perceived competence.

### 6.4. Implications for Professional Development

A central finding of this study is that the two dimensions of ICT implementation showed different predictor patterns. While pedagogical ICT activities were predicted by both equipment and self-efficacy, ICT use with children was predicted only by self-efficacy and migration background. This differentiation underscores that ICT implementation is not a unitary construct and that different aspects of implementation may require different forms of support. It also highlights the need for further research into what constitutes effective ICT implementation in ECEC and how its various dimensions relate to children’s learning outcomes.

Results suggest that strengthening educators’ ICT-related self-efficacy is at least as important as providing access to devices. Professional development should therefore go beyond technical training and include opportunities for practical experience in planning and conducting ICT-based learning activities with children, as well as critical reflection on the educational value of ICT. Investment in digital infrastructure by providers and professional development organizations requires support for educators’ ICT-related competencies at the same time to transfer them into meaningful educational ICT integration. Additionally, the finding that self-efficacy predicted both outcomes points to the potential value of formats that allow educators to experience success in ICT-related educational situations, such as peer coaching, mentoring, or guided practice within the team (von Suchodoletz et al., 2018). At the same time, self-efficacy alone is unlikely to be sufficient for high-quality ICT implementation. Perceived competence must be grounded in actual pedagogical and technological knowledge to result in meaningful practice. Professional development programs should therefore combine competence-building elements that strengthen self-efficacy with knowledge-oriented components that equip educators with the pedagogical and technical understanding necessary to plan and evaluate developmentally appropriate ICT-based activities (Ertmer & Ottenbreit-Leftwich, 2010; Redecker, 2017). These findings should be interpreted considering the broader debate about ICT in early childhood and education. Health organizations such as the WHO (2019) and the American Academy of Pediatrics (2016) recommend limiting young children’s screen exposure. These recommendations, however, primarily concern passive and unsupervised consumption, not guided pedagogical use. Organizations in the field of early childhood education have taken a different position and emphasize that technology can support learning when it is intentional and developmentally appropriate (NAEYC & Fred Rogers Center, 2012). The present results are consistent with this distinction. It was not beliefs about ICT that predicted implementation but perceived competence to use it purposefully. Educators who are skeptical about digital media in early childhood are not necessarily less effective. Their concerns can be grounded in legitimate pedagogical considerations about developmental appropriateness (Mertala, 2019; Palaiologou, 2016). Rather than trying to overcome critical attitudes, professional development should support educators in identifying when and how ICT use can be meaningful for young children.

Further, the finding that centers serving children with migration backgrounds showed lower ICT use with children is relevant for professional development and institutional support programs as well.

## 7. Limitations and Future Directions

The cross-sectional design does not allow causal conclusions. It is not possible to determine whether ICT-related beliefs and self-efficacy lead to greater ICT implementation, or whether experience with ICT in practice strengthens these orientations over time. Longitudinal studies and evaluations of professional development interventions with pre–post measurement are needed to address this.

Self-selection effects cannot be excluded, and educators with a stronger interest in digital technologies may be overrepresented. Findings should therefore be interpreted with caution regarding generalizability. It should further be noted that data collection coincided with the COVID-19 pandemic, which may have affected both ICT infrastructure in centers and educators’ technology use patterns (Cohen et al., 2021).

Further, both outcomes relied on educator self-reports. Observational measures or experience-sampling methods documenting actual ICT activities with children would provide more ecologically valid data.

Explained variance was modest across all models (R^2^ = .047–.144), suggesting that relevant predictors were not captured in this study.

Unmodelled factors likely include team characteristics, leadership culture around ICT, availability of technical and pedagogical support, and participation in ICT-specific professional development, which may shape individual educators’ ICT-related orientations and practices. The present study focused on the professional competencies themselves, specifically beliefs, self-efficacy, and affective–motivational orientations, rather than on the formal pathways through which these competencies are acquired (Baumert & Kunter, 2011). Whether and how leadership support and professional development contribute to the formation of ICT-related self-efficacy remains an important question for future research.

The operationalization of ICT equipment as a count index of device types does not distinguish between the number of devices available, their quality, or their actual accessibility during educational activities. Future studies should examine whether it is the variety of available device types, the number of devices, or the availability of multifunctional devices in particular that matters most for implementation. A related question is whether more equipment is necessarily better, or whether a smaller number of high-quality multifunctional devices that are accessible to educators and children during activities may lead to more meaningful ICT integration than a large but fragmented set of devices.

A related limitation concerns the operationalization of *ICT use with children* as a summative index of usage frequency across six device types. This variety-based measure targets two different concepts. First, routine use distributed across multiple devices and second, intentional, educationally focused use might concentrate on a small number of multifunctional devices. As a result, educators who deploy a single tablet as a simultaneous camera, production, and documentation tool may score lower on this index than educators who use several devices infrequently and without explicit educational intent. This limits the construct validity of the measure. Future studies should distinguish frequency of use, breadth of device types, and intentionality as separate dimensions of ICT implementation.

A further measurement concern relates to the ICT use with children index. Because the frequency rating for each device type is bounded by its availability, educators who lack a particular device automatically receive the lowest possible frequency rating for that device. The index therefore conflates non-availability with non-use, which may produce floor effects and restrict variance. This confound likely contributes to the low observed mean (M = 3.89 out of a theoretical maximum of 36) and may attenuate associations with predictor variables.

Both outcomes measure the frequency of ICT use or activities but do not capture the pedagogical quality of these interactions. This points to a broader conceptual limitation concerning the normative assumption underlying measurements of ICT use, namely the assumption that more is better. Whether more frequent ICT use or pedagogical ICT activities actually promote children’s development depends on the quality of these interactions, not their quantity. In this context, it also remains an open empirical question whether engagement with ICT opens up new educational opportunities for children or displaces or replaces other beneficial activities. Future research should examine not only frequency measurements but also the qualitative dimensions of ICT implementation.

Finally, findings are situated within the German ECEC system. Cross-national replication is needed to determine which associations hold across different regulatory frameworks, professional cultures, and infrastructure conditions.

## 8. Conclusions

The present study investigated how structural characteristics and educators’ professional competencies predict ICT implementation in ECEC. Two outcomes were examined, ICT use with children and pedagogical ICT activities, and both showed different patterns. The ICT equipment in the center was associated with pedagogical ICT activities but not with the general use of devices. Self-efficacy was associated with both outcomes. Centers with higher proportions of children with a migration background showed lower levels of ICT use together with children. These results indicate that different aspects of ICT implementation depend on different conditions. Both material resources and professional competencies matter, but self-efficacy appears consistently as a predictor for both dimensions. Longitudinal research is needed to clarify the direction of these associations and to evaluate whether professional development that strengthens ICT-related self-efficacy leads to sustained changes in pedagogical practice.

## Figures and Tables

**Table 1:** Descriptive statistics and reliability coefficients of the variables.

Variable	M	SD	α	*n*
*Outcome variables*				
1. ICT use with children	3.89	1.14	—	249
2. Pedagogical ICT activities	4.30	2.28	—	227
*Predictor variables*				
3. ICT beliefs	2.78	0.47	.91	219
4. ICT self-efficacy	2.84	0.70	.81	218
5. ICT enthusiasm	3.00	0.75	.87	218
6. ICT anxiety	1.47	0.57	.77	218
*Control variables*				
7. ICT equipment in the center	2.68	1.46	—	258
8. Proportion of children with migration background (%)	32.80	28.90	—	253
9. WLAN availability (%)	66.00	—	—	258
1. Staff–child ratio	5.77	2.47	—	255
11. Children under three (%)	23.96	30.21	—	255

Note. Dashes in the α column indicate formative indices for which internal consistency is not an applicable reliability criterion. *n* = number of valid observations per variable.

**Table 2:** Intercorrelations between the variables.

Variable	1	2	3	4	5	6	7	8	9	10	11
1. ICT use with children	—										
2. Pedagogical ICT activities	.22 *	—									
3. ICT beliefs	.28 **	.31 **	—								
4. ICT self-efficacy	.25 **	.22 **	.23 **	—							
5. ICT enthusiasm	.18 *	.15 *	.60 **	.53 **	—						
6. ICT anxiety	−.18 *	−.15 *	−.12 †	−.35 **	−.35 **	—					
7. ICT equipment in the center	.19 *	.16 *	.05	.02	.01	−.06	—				
8. Proportion of children with migration background	−.21 *	−.24 **	.05	−.02	.02	.03	.05	—			
9. WLAN availability	−.01	.08	−.09	−.07	−.13 †	−.02	.27 **	−.00	—		
1. Staff–child ratio	−.06	−.03	−.09	−.08	−.08	.06	−.07	−.08	.04	—	
11. Children under three (%)	.02	−.01	.02	−.02	.02	−.06	−.02	−.13 *	−.05	−.39 **	—

Note. N = 211–258 depending on variable pair due to pairwise deletion of missing data. Correlations are Pearson’s r. † *p* < .10. * *p* < .05. ** *p* < .01.

**Table 3:** Hierarchical regression predicting ICT use with children.

Predictor	Model 1	Model 2	Model 3
*Block 1: Structural characteristics*			
WLAN availability	.02	.01	.04
ICT equipment in the center	−.10	−.09	−.10
Proportion of children with migration background	−.20 **	−.19 **	−.18 *
Staff–child ratio	−.11	−.11	−.09
Children under three (%)	−.05	−.05	−.04
*Block 2: Beliefs*			
ICT beliefs		−.07	−.17 †
*Block 3: Motivational orientations*			
ICT self-efficacy			.19 *
ICT enthusiasm			.08
ICT anxiety			−.06
R^2^	.050	.055	.116
N	266	266	266

Note. Standardized regression coefficients (β) are reported. † *p* < .1. * *p* < .05. ** *p* < .01.

**Table 4:** Hierarchical regression predicting pedagogical ICT activities.

Predictor	Model 1	Model 2	Model 3
*Block 1: Structural characteristics*			
WLAN availability	.03	.05	.08
ICT equipment in the center	.17 *	.16 *	.16 *
Proportion of children with migration background	−.12 †	−.13 *	−.12 †
Staff–child ratio	−.05	−.03	−.01
Children under three (%)	−.03	−.03	−.00
*Block 2: Beliefs*			
ICT beliefs		.20 **	.10
*Block 3: Motivational orientations*			
ICT self-efficacy			.20 *
ICT enthusiasm			.12
ICT anxiety			.13
R^2^	.047	.087	.144
N	266	266	266

Note. Standardized regression coefficients (β) are reported. † *p* < .1. * *p* < .05. ** *p* < .01.

## Data Availability

The data supporting the findings of this study are openly available at GESIS—Leibniz Institute for the Social Sciences (Cohen & Anders, 2023). https://doi.org/10.7802/2553.

## Note

1 In this study, digital technologies or ICT refer to digital devices and applications used within educational contexts, including tablets, computers, cameras, and digital applications that may support children’s learning and exploration (Plowman, 2016).
